# Content validation of the EORTC QLQ-C15-PAL with advanced cancer patients and health care professionals from palliative care services in Chile

**DOI:** 10.1186/s12904-020-00586-1

**Published:** 2020-06-09

**Authors:** Leslye Rojas-Concha, Maiken Bang Hansen, Morten Aagaard Petersen, Mogens Groenvold

**Affiliations:** 1 The Palliative Care Research Unit, Department of Geriatrics and Palliative Medicine GP, Bispebjerg and Frederiksberg Hospital, University of Copenhagen, Bispebjerg Bakke 23, DK-2400 Copenhagen, NV Denmark; 2 Department of Public Health, University of Copenhagen, Copenhagen, Denmark

**Keywords:** EORTC QLQ-C15-PAL, Advanced cancer, Palliative care, Symptom assessment, Quality of life, Patient-reported outcomes

## Abstract

**Background:**

The assessment of patients’ quality of life (QOL) is essential when evaluating the outcome of palliative care; however, no instruments have been validated for measuring symptoms and QOL in patients receiving palliative care in Chile. We aimed to investigate the content validity of the EORTC Quality of Life Questionnaire Core 15 Palliative Care (QLQ-C15-PAL), replicating the methods used previously to shorten the EORTC Quality of Life Questionnaire Core 30 (QLQ-C30) for use among patients in palliative care.

**Methods:**

This cross-sectional study was conducted from October to November 2017 in four palliative care services. Patients with advanced cancer and health care professionals (HCPs) were invited to individual interviews to determine the relevance, appropriateness and relative importance of the 30 items of the QLQ-C30 for evaluating the outcome of palliative care, and whether relevant additional issues should be included.

**Results:**

A total of 48 patients and 35 HCPs participated. The most important dimensions selected were pain, physical functioning, sleeping difficulties, emotional functioning, nausea/vomiting, fatigue, social functioning, lack of appetite, role functioning and constipation. Qualitative data identified important additional issues not covered by the questionnaire such as satisfaction with care, emotions and psychological support, as well as linguistic issues in the dyspnea and constipation items.

**Conclusions:**

The EORTC QLQ-C15-PAL showed good content validity in the assessment of symptoms and QOL of advanced cancer patients; therefore, we recommend the use of this questionnaire in palliative care in Chile. Dyspnea and constipation items were revised by the EORTC group. More research is needed to add a social dimension for a comprehensive assessment of patients’ QOL.

## Background

Palliative care aims to improve the quality of life (QOL) of patients through the early detection and treatment of their symptoms [1]. Thus, the assessment of patients’ QOL is essential when evaluating the outcome of palliative care. Nevertheless, assessment of QOL may be difficult in patients with advanced cancer due to the number and severity of symptoms they experience. To reduce the burden on patients, patient-reported outcomes (PROs) instruments in palliative care should be brief, cover the main symptoms and problems, and at the same time avoid content of limited relevance.

The European Organization for Research and Treatment of Cancer (EORTC) Quality of Life Questionnaire Core 15 Palliative Care (QLQ-C15-PAL) is an abbreviated 15-item version of the EORTC Quality of Life Questionnaire Core 30 (QLQ-C30), which is one of the most widely used, validated, translated and published instruments for assessing symptoms and QOL in cancer patients [2]. The QLQ-C15-PAL contains the items of the QLQ-C30 which were identified as the most important for palliative care, based on interviews with health care professionals and patients [3], and on statistical analyses using item response theory [4, 5]. This development process secures that studies may compare their results obtained from the QLQ C15-PAL with studies using the original QLQ-C30 questionnaire [3]. In addition, the QLQ-C15-PAL has been successfully validated and translated in several countries [6–14], including Spanish-speaking countries [15, 16].

The Chilean-Spanish version of the QLQ-C30 and some modules have been validated before [17, 18]. A few studies have used the QLQ-C30 to evaluate patients’ QOL in oncology research [19–22], but no instruments have been validated for measuring symptoms and QOL in patients receiving palliative care in Chile. When the QLQ-C15-PAL was developed, its content validity was evaluated by health care professionals and patients from six European countries [3]. However, because of differences between these European countries and Chile in their health care systems, including how palliative care is organized, and the availability of palliative care services, it would be relevant to investigate the content validity of the QLQ-C15-PAL in Chilean population. In Chile most palliative care services are in the public health care system, but some services are fully private. Although Chile has the highest number of palliative care services in Latin America [23], Chile still has a fewer number of palliative services compared to the Western European countries. In 2013, the ratio of services to the population in Chile was 1: 808,000, which is lower compared to the six European countries with a ratio of services to the population between 1: 48,000 (in the United Kingdom), and 1: 122,000 (in Denmark) [24]. Therefore, we aimed to investigate the content validity of the QLQ-C15-PAL questionnaire with patients and health care professionals from palliative care services in Chile, replicating the methods used previously to shorten the items of the QLQ-C30 among European patients in palliative care [3].

## Methods

### Patients and health care professionals

This cross-sectional study was conducted from October to November 2017 with participants from the palliative care services of four public hospitals in Santiago in Chile. We planned to recruit at least 10 patients and 10 heath care professionals (HCPs) from each palliative care service.

Outpatients with advanced cancer undergoing palliative care treatment, with knowledge of their diagnosis, and who had given informed consent were eligible. Patients who were too ill to participate in interviews, and non-native Spanish speakers were excluded. HCPs with their main job in palliative care were eligible. For patients, the primary cancer site was registered, occupation and years of experience were registered for HCPs, and demographic characteristics were collected for all participants.

### Questionnaires

The Chilean-Spanish version of the QLQ-C30 questionnaire (version 3) was used for this study, and was obtained from the EORTC Quality of Life Department in Brussels. The QLQ-C30 consists of 30 items distributed in five functional scales measuring physical, emotional, role, cognitive and social functioning; three symptoms scales measuring fatigue, pain, nausea/vomiting; one scale measuring “global health and quality of life status”, and six single items measuring sleeping difficulties, dyspnea, constipation, lack of appetite, financial difficulties, and diarrhea. All items are rated on 4-points Likert scales that range from 1 (not at all) to 4 (very much), with the exception of the global health/quality of life scale, which is rated from 1 (very poor) to 7 (excellent) [25, 26].

The QLQ-C15-PAL is an abbreviated version of the QLQ-C30, which was developed for patients in palliative care preserving the main advantages of the original questionnaire. The QLQ-C30 was shortened to 15 items by omitting items of the QLQ-C30 that were considered inappropriate or not highly relevant in palliative care by patients and health care professionals in interviews, [3] and by shortening multi-item scales of QLQ-C30 using item response theory [4, 5]. Four scales, i.e., physical functioning, emotional functioning, nausea/vomiting and fatigue, were shortened retaining the items that best predicted scores on the original scales. Due to the low relevance reported in the interviews, the two-item global QOL scale was reduced to one item by omitting overall health, and five scales/items were completely omitted (social, role and cognitive functioning scales, diarrhea and financial difficulties items).

### Procedure for interviews

To validate the content of the QLQ-C15-PAL questionnaire, we conducted semi-structured interviews following the procedure originally used to develop a shortened version of the QLQ-C30 for palliative care patients [3], and the EORTC Quality of Life Group Guidelines for developing questionnaire modules [27]. The purpose of these interviews was to identify the most relevant issues for patients in palliative care among those included in the EORTC QLQ-C30 in order to investigate whether the QLQ-C15-PAL also has the appropriate content for use in Chile.

Patients were asked to respond to the Chilean-Spanish version of the QLQ-C30 during individual interviews by a trained researcher. The interviewer then invited patients to comment on how well the QLQ-C30 items “evaluated the outcome of the palliative care they received”. Patients were asked to comment on the QLQ-C30 items in terms of relevance, appropriateness, relative importance, and breadth of coverage. The relevance of each item was rated using the response categories 1 “not at all”, 2 “a little”, 3 “quite a bit” and 4 “very much”. If an item was rated 1 or 2, patients were asked to report the reasons, e.g., “Why do you consider this question not or only a little relevant?”. The appropriateness was measured asking patients to identify items they perceived as inappropriate or upsetting. The relative importance was evaluated asking patients to select the 10 most important items when evaluating the success of palliative care. The breadth of coverage was assessed asking patients to report additional issues that were relevant for them but not covered by the QLQ-C30.

An identical interview was followed for HCPs, but they were required to comment on how well the QLQ-C30 items “evaluated the outcome of palliative care in general”, i.e., not for a specific patient, but for patients admitted to palliative care in general. HCPs were asked to comment on relevance, appropriateness, relative importance, and breadth of coverage of the QLQ-C30 items, using the same procedure as described above.

### Statistical analysis

Participants’ characteristics were expressed as proportions for categorical variables, and as means, standard deviations, medians and ranges for continuous variables. The mean relevance score of each item was calculated after transforming the responses to 0–100 scales with 0 corresponding to “not at all” and 100 to “very much” [26]. The proportion of participants rating each item as “inappropriate or upsetting” and selecting each item as one of the 10 most important were calculated. The proportion of participants who selected at least one of the items from each multi-item scale as one of the 10 most important was also calculated. Single items and multi-item scales were ranked according to the proportion of patients and HCPs selecting them as among the 10 most important. This ranking named ‘importance percentage’ was calculated as the average: (percentage of patients + percentage of HCPs)/2; although the sample of patients was larger than HCP sample, both received equal weight. Furthermore, this ranking was used to compare the relative importance of the scales and items as perceived by Chilean patients/HCPs with the results of the original study [3]. HCPs were divided in two subgroups, i.e., “physicians” and “other HCPs” to explore differences between the rating of relevance and importance for each dimension of the QLQ-C30, tested by Mann-Whitney U Test. Qualitative data obtained from the interviews were categorized into responses about the lack of relevance in selected items, and the need to include other issues in the questionnaire, respectively. A *p* value < 0.05 was considered statistically significant. Analyses were performed using the statistical software Statistical Package for the Social Sciences (SPSS) version 23.

## Results

### Participation

A total of 48 patients with advanced cancer and 35 HCPs participated in the interviews. The median age of patients was 60 years, and the most common cancer diagnoses were prostate cancer (14.6%), stomach cancer (10.4%) and multiple myeloma (10.4%). HCPs had a median age of 39 years and the majority were physicians (34.3%), followed by nurses (20.0%), and psychologists (20.0%). For further details see Table 1.

**Table 1:** Characteristics of 48 patients and 35 health care professionals participating in interviews

Patient characteristics	Patients		Health care professionals	
	*N*	(%)	*N*	(%)
Sex				
Men	16	(33.3)	9	(25.7)
Women	32	(66.7)	26	(74.3)
Age				
Mean (SD)	59.2	(13.1)	40.9	(12.7)
Median (range)	60	(29–86)	39	(23–70)
Hospitals				
Sotero del Rio	27	(56.3)	12	(34.3)
San Juan de Dios	4	(8.3)	5	(14.3)
Salvador	13	(27.1)	11	(31.4)
Felix Bulnes Cerda	4	(8.3)	7	(20.0)
Diagnosis (cancer site, ICD-10)				
Prostate (C61)	7	(14.6)		
Stomach (C16)	5	(10.4)		
Multiple myeloma (C90)	5	(10.4)		
Breast (C50)	4	(8.3)		
Colorectal (C18-C20)	3	(6.3)		
Melanoma skin cancer (C43)	3	(6.3)		
Uterine (C54-C55)	3	(6.3)		
Ovarian (C56, C570-C574)	3	(6.3)		
Liver (C22)	2	(4.2)		
Sarcoma (C46-C49)	2	(4.2)		
Cervical (C53)	2	(4.2)		
Kidney (C64-C66)	2	(4.2)		
Lymphoma (C81-C85)	2	(4.2)		
Other cancer (all other C codes)	5	(10.4)		
Discipline of HCPs				
Physician			12	(34.3)
Nurse			7	(20.0)
Psychologist			7	(20.0)
Paramedic			6	(17.1)
Physiotherapist			1	(2.9)
Social worker			1	(2.9)
Nutritionist			1	(2.9)
Years of experience of HCPs				
Mean (SD)			13.9	(11.9)
Median (range)			11	(1–42)

*SD* standard deviation, *ICD-10* International Statistical Classification of Diseases and Related Health Problems 10th Revision

### Interviews

#### Relevance, appropriateness and relative importance of the EORTC QLQ-C30

The quantitative data including ratings of relevance, inappropriateness, and relative importance of items are summarized in Table 2, together with the qualitative data from categorized responses to the question: “Why do you consider this question not or only little relevant?” presented in Table 3, will be discussed simultaneously for each dimension of the QLQ-C30 questionnaire. Scales and items are presented in the order in which they appear in Tables 2 and 3, i.e., based on their importance rating, where the 10 most important were pain, physical functioning, sleeping difficulties, emotional functioning, nausea/vomiting, fatigue, social functioning, lack of appetite, role functioning and constipation.

**Table 2:** Relevance, inappropriateness and importance of items reported by 48 patients and 35 health care professionals

Scale/item	Item	Relevance (mean)		% Inappropriate		% Selected as one of the most important		
		Pts.	HCPs	Pts.	HCPs	Pts.	HCPs	/2^a^
Pain (PA)	**9**	100	97	0	0	96	77	
	**19**	97	80	2	6	27	54	
Any PA item						98	94	96
Physical functioning (PF)	1	77	64	6	9	40	3	
	2	78	63	2	0	25	3	
	**3**	76	73	4	3	17	9	
	**4**	83	85	6	3	23	51	
	**5**	98	95	0	0	60	71	
Any PF item						98	80	89
Sleeping difficulties (SL)	**11**	97	95	0	0	75	86	80
Emotional functioning (EF)	**21**	95	63	0	3	19	6	
	22	93	68	0	2	21	11	
	23	92	73	0	0	21	17	
	**24**	98	89	0	0	38	60	
Any EF item						73	83	78
Nausea and vomiting (NV)	**14**	96	88	0	0	25	54	
	15	97	87	0	0	35	46	
Any NV item						46	69	57
Fatigue (FA)	10	97	67	2	3	50	6	
	**12**	92	73	0	3	31	20	
	**18**	92	74	2	3	42	14	
Any FA item						77	34	56
Social functioning (SF)	26	93	89	2	3	31	54	
	27	88	83	4	0	8	31	
Any SF item						38	60	49
Lack of appetite (AP)	**13**	99	86	0	0	35	57	46
Role functioning (RF)	6	95	79	0	0	50	23	
	7	82	57	2	6	31	6	
Any RF item						60	26	43
Constipation (CO)	**16**	92	83	0	0	29	54	42
Financial difficulties (FI)	28	93	81	4	3	35	49	42
Global health status/	29	97	90	0	0	8	23	
Quality of life (QOL)	**30**	95	89	0	3	15	49	
Any QL item						23	54	39
Dyspnea (DY)	**8**	70	70	2	3	46	17	31
Cognitive functioning (CF)	20	90	54	0	3	21	6	
	25	97	67	0	0	15	14	
Any CF item						33	17	25
Diarrhea (DI)	17	93	79	2	0	17	20	18

The scales/items are ranked according to importance. Items in bold form were extracted from the EORTC QLQ-C30 to form the EORTC QLQ-C15-PAL questionnaire

^a^ The mean of the values for patients (Pts.) and health care professionals (HCPs)

**Table 3:** Categorized reasons why some items were rated as little or not relevant by the participants

Scale/item	Item	Relevance^a^		Technical issues^b^		Inappropriate^c^		Relative^d^		Not important^e^		Difficult^f^		Total
		Pts.	HCPs	Pts.	HCPs	Pts.	HCPs	Pts.	HCPs	Pts.	HCPs	Pts.	HCPs	
Pain	9				1									1
	19		3		1		2	1						7
Physical functioning	1	5	5			3	3	1						17
	2	4	6	1	1	1		1		1	1			16
	3	4	6	1	2	1	1	1		1				17
	4	3				2	1	1					1	8
	5													–
Emotional functioning	21	1	5	1	2		1		1		1	1		13
	22	1	5	2	1			1		1	1			12
	23		1	4	5			1			1			12
	24		2		3			1						6
Fatigue	10		5		1		1	1		1	1			10
	12		3	2	3		1	2					1	12
	18	1	4	1			1	2			1			10
Nausea and vomiting	14	1	2		2									5
	15		2		1									3
Sleeping difficulties	11				1									1
Social functioning	26	1				1	1	1			1			5
	27		1		2	2		1			2			8
Dyspnea	8	3	5	14	13	1		1		1		1		39
Role functioning	6	2	1		3						1			7
	7	4	11		1	1	2	1						20
Constipation	16		1	10	7									18
Lack of appetite	13		1											1
Financial difficulties	28	1	2		1	2	1					1		8
Global health status/	29		2	1									1	4
Quality of life	30		1	2	3		1	1				1	1	10
Cognitive functioning	20	4	11		1		1				4			21
	25	1	8		1				1					11
Diarrhea	17		3			1			1					5
Total		36	96	39	56	15	17	18	3	5	14	4	4	307

##### Pain (PA) scale

Pain was selected as the most important dimension of the QLQ-C30 and both items were rated as highly relevant.

##### Physical functioning (PF) scale

The last item 5 “need help with self-care” was rated as the most important item of the scale, followed by item 4 “stay in bed”. Items 2 “long walk” and 3 “short walk” were rated as less relevant by the respondents, who mentioned that these questions regarding to walking limitations were not appropriate for patients in palliative care. Item 1 “strenuous activities” was the item most often rated as inappropriate by HCPs (9%).

##### Sleeping difficulties (SL) item

Sleeping difficulties was rated as very relevant and chosen as one of the most important dimensions by 80% of participants.

##### Emotional functioning (EF) scale

The four items of this scale were rated more relevant by patients than by HCPs; however, 83% of HCPs compared to 73% of patients selected this scale as one of the 10 most important for palliative care. Item 24 “feel depressed” was most often selected as important particularly by HCPs. Nine participants indicated that item 23 “feel irritable” was poorly formulated, e.g., the word “irritable” could be replaced by “angry” (Table 3).

##### Nausea and vomiting (NV) scale

Nausea and vomiting were rated as highly relevant by respondents and selected as two important items by 69% of the HCPs.

##### Fatigue (FA) scale

More than a half of the patients selected fatigue as an important dimension (77%) in comparison with the HCPs (34%). Item 12 “feel weak” was the least important item selected by HCPs, mainly because they believed that this symptom was repeatedly measured. HCPs found that item 10 “need to rest” was the least important and less relevant of the scale.

##### Social functioning (SF) scale

Social functioning scale was selected as an important dimension by 49% of the participants. Both items, item 26 “your physical condition or medical treatment has interfered with your family life” and item 27 “your physical condition or medical treatment has interfered with your social activities” were scored with high relevance, especially item 26.

##### Lack of appetite (LA) item

Lack of appetite was more selected as an important item by HCPs than by patients (57% vs. 35%).

##### Role functioning (RF) scale

Role functioning scale was very important for patients (60%), scoring with a high relevance the item 6 “limitations at work or daily activities”.

##### Constipation (CO) item

Constipation was considered by 42% of the respondents as an important item, and about 20% (*n* = 17) of them suggested linguistic changes for this question because it was difficult to understand by patients (See also Table 4).

**Table 4:** Categorized comments about linguistic issues found in the dyspnea and constipation items of EORTC QLQ-C30

Scale/item	Item	Participant comments and suggestions for alternative wording (quotation marks)	Pts.	HCPs
Dyspnea	8	Not well formulated	1	2
		“Ran out of air”	1	2
		“Lack of air”	3	4
		“Ran out of breath”	5	2
		“Difficulty breathing”	2	1
		“Maximum tiredness”		2
		Did not understand “short of breath”	2	
		Total	14	13
Constipation	16	“Difficulty defecating”	3	6
		“Troubles defecating”	2	1
		“Bowel movements”	1	2
		Did not understand “constipated”	1	1
		Total	7	10

*Pts* Patients, *HCPs* health care professionals

##### Financial difficulties (FI) item

This item was chosen as one of the most important by 49% of the HCPs compared to 35% of the patients. Few participants described this item as inappropriate for palliative care setting.

##### Global health status/ quality of life (QOL) scale

Participants rate both items as highly relevant. This dimension was selected as one of the most important more often by the HCPs than by patients (54% vs. 23%), although some respondents reported that item 30 as little relevant because they believed the concept “quality of life” is not understood by all patients in palliative care.

##### Dyspnea (DY) item

Similar numbers of patients and HCPs reported that dyspnea was a not well formulated item making it difficult for patients to comprehend, whereas 33% of participants (*n* = 27) suggested linguistic changes for dyspnea (Table 4).

##### Cognitive functioning (CF) scale

Cognitive functioning was generally regarded as less important than the other five functioning scales, selected only by 25% of respondents. Item 20 “concentrating problems” was the least relevant item in this scale.

##### Diarrhea (DI) item

Although rated as relevant, diarrhea was the issue least often selected as important.

The comparison of item relevance and importance scores between physicians (*n* = 12) and other HCPs (*n* = 23) showed no significant differences; therefore, they are not shown in Table 2.

#### Breadth of coverage of the EORTC QLQ-C30

Patients and HCPs were asked to report additional issues that were not included in the QLQ-C30, which they considered relevant for the outcome of the palliative care. In total, 91 topics were mentioned by the respondents. These were grouped into 10 overalls categories. The three most frequent categories were satisfaction with care, emotions and psychological support. Satisfaction with care included topics about satisfaction of patients with HCPs, and effectiveness of medication, mostly reported by patients. Emotions included topics about role loss and mood changes, and psychological support included phycological needs, and facing life with advanced cancer. For further details see Table 5.

**Table 5:** Additional issues that would be relevant to include when evaluating the outcome of palliative care

Additional issues categories	Pts.	HCPs
Satisfaction with care	8	4
Satisfaction with health care professionals, satisfaction with the information received, adherence to treatment, effectiveness of medication and side effects		
Emotions	5	7
Role loss, mood changes, sadness, anhedonia, fear		
Psychological support	6	6
Psychological needs, significant changes to the way of living, facing life with advanced cancer, measure psychological distress, personality disorders		
Sexuality	1	10
Sexual satisfaction, sexual activity		
Social support	4	5
Support from family/relatives, caregivers’ distress, cohabitation, e.g., whom do you live with?		
Symptoms and problems	2	7
Visual problems, sleeping tongue, eating/swallowing problems, drowsiness, dementia/delirium, urinary problems		
Existential issues	1	8
Thoughts about death, uncertainty about future, transcendence		
Spiritual issues		6
Spiritual pain, spirituality		
Physical difficulties	3	3
Ability to move around on you own at home, toileting independence		
Economic problems	1	4
Delay in sick leave payment, transportation expenses, e.g., go to hospital		
Total number of issues	31	60

*Pts* Patients, *HCPs* health care professionals

## Discussion

In this study, we performed a content validation of the QLQ-C15-PAL with 48 patients and 35 HCPs from four palliative care services in Chile, replicating the methodology of a previous study conducted in six European countries [3]. In general, our results were similar to that study, confirming the content validity of the QLQ-C15-PAL questionnaire, but we made important observations that will be discussed later.

Of the 10 function/symptom scales included in the QLQ-C15-PAL questionnaire, eight were selected among the 10 most important dimensions to include in the assessment of palliative care in Chile, i.e., pain, physical functioning, sleeping difficulties, emotional functioning, nausea/vomiting, fatigue, lack of appetite and constipation. Responses about the five shortened scales from the original QLQ-C30 to form the QLQ-C15-PAL were comparable to the Groenvold et al. study [3]. Physical functioning and fatigue scales were particularly important scales by patients, whereas emotional functioning, nausea/vomiting and global health status/QOL scales were essential for HCPs. Most of the items of these scales that were finally retained in the QLQ-C15-PAL, were also selected by our respondents as relevant issues to measure the outcome of palliative care.

The most important dimension selected by the participants was pain (96%), which has been recognized in the literature as one of the most prevalent symptoms reported by advanced cancer patients in palliative care [28, 29]. Other dimensions frequently selected as important were physical functioning (89%), sleeping difficulties (80%), emotional functioning (78%), nausea/vomiting (57%), fatigue (56%) and social functioning (49%). These dimensions were previously identified as prevalent palliative needs in a study investigating the content validity of PROs instruments in palliative care, by comparing patient reported symptoms and problems to what was registered in the medical records [30]. Although social and role functioning were excluded in the development of the QLQ-C15-PAL due to the lack relevance reported in the original study [3], in our study these dimensions were selected as the most important by 49 and 43% of respondents respectively, principally HCPs selected social functioning in relation to family life of patients, and patients selected role functioning in relation to their limitations at work. Patients’ concern about their role in the family, the social support they received from family, and personal challenges related to work have been reported before by a palliative care service in a small qualitative study conducted in Chile [31].

Qualitative data corresponding to additional issues not covered by the questionnaire showed that HCPs reported twice as many topics as patients did. A reason may be that HCPs have the perspective of many patients while patients focus on their own situation. Further, the breadth of coverage question was asked at the end of the interview, hence, some patients may have been too fatigued to give comprehensive responses. The main additional issues reported in our study were satisfaction with care, emotions and psychological support. In contrast, additional issues related with existential and spiritual issues were frequently reported by Groenvold et al. [3]. Further research is needed to evaluate which aspects not covered by the QLQ-C15-PAL may be relevant for a comprehensive measurement of the QOL in Chilean palliative care patients, e.g., a social dimension. While social support for patients in palliative care and their families has been recommended by the Chilean Ministry of Health [32], as well as international organizations [1, 33], social needs do not seem to be covered by current palliative care in Chile, as only one of the four palliative care services in this study had a social worker in their teams.

The qualitative data was useful to identify unexpected linguistics issues in the dyspnea and constipation items, since 33 and 20% of the participants reported that these questions were not well formulated or were difficult to understand. A list with the comments about these two items was submitted to the Translation Unit of the EORTC Quality of Life Department for possible revision of the translation of these items. After their analysis both items were modified in the Chilean versions of the QLQ-C30, and the QLQ-C15-PAL questionnaires.

We recognize some limitations related to this study. First, we did not evaluate statistically the psychometric properties of the QLQ-C15-PAL in Chilean patients; however, it has been extensively validated in previous international studies [6–13]. Second, we planned to recruit at least 10 HCPs from each palliative care service, but half of the services investigated had less than 10 professionals in their teams. Nevertheless, we had no missing data for the study analysis, since the participants were accompanied by the researcher during their self-assessment of the QLQ-C30 or were assisted if necessary.

## Conclusions

The EORTC QLQ-C15-PAL showed good content validity in the assessment of symptoms and QOL of advanced cancer patients. Additionally, we identified linguistic issues in the dyspnea and constipation items that were revised by the EORTC group. This questionnaire may help clinicians, and researchers to initiate palliative care interventions that may improve QOL of patients. Therefore, we recommend the use of the EORTC QLQ-C15-PAL in patients receiving palliative care in Chile. More research is needed to add a social dimension for a comprehensive assessment of patients’ QOL in Chile.

## Data Availability

The datasets used and/or analyzed during the current study are available from the corresponding author on reasonable request.

## Abbreviations

QOL: Quality of life
QLQ-C15-PAL: The European Organization for Research and Treatment of Cancer Quality of Life Questionnaire Core 15 Palliative Care
QLQ-C30: The European Organization for Research and Treatment of Cancer Quality of Life Questionnaire Core 30
HCPs: Health care professionals
Pts.: Patients
PROs: Patient-reported outcomes

## References

[1] Sepúlveda C, Marlin A, Yoshida T, Ullrich A (2002). Palliative care: the World Health Organization’s global perspective. J Pain Symptom Manag.

[2] Velikova G, Coens C, Efficace F, Greimel E, Groenvold M, Johnson C, Singer S, van de Poll-Franse L, Young T, Bottomley A (2012). Health-related quality of life in EORTC clinical trials — 30 years of progress from methodological developments to making a real impact on oncology practice. EJC Suppl.

[3] Groenvold M, Petersen MA, Aaronson NK, Arraras JI, Blazeby JM, Bottomley A, Fayers PM, de Graeff A, Hammerlid E, Kaasa S (2006). The development of the EORTC QLQ-C15-PAL: a shortened questionnaire for cancer patients in palliative care. Eur J Cancer.

[4] Petersen MA, Groenvold M, Aaronson N, Blazeby J, Brandberg Y, de Graeff A, Fayers P, Hammerlid E, Sprangers M, Velikova G (2006). Item response theory was used to shorten EORTC QLQ-C30 scales for use in palliative care. J Clin Epidemiol.

[5] Bjorner JB, Petersen MA, Groenvold M, Aaronson N, Ahlner-Elmqvist M, Arraras JI, Bredart A, Fayers P, Jordhoy M, Sprangers M (2004). Use of item response theory to develop a shortened version of the EORTC QLQ-C30 emotional functioning scale. Qual Life Res.

[6] Shin DW, Choi JE, Miyashita M, Choi JY, Kang J, Baik YJ, Mo HN, Park J, Kim H-J, Park EC (2011). Cross-cultural application of the Korean version of the European Organization for Research and Treatment of Cancer quality of life questionnaire-Core 15-palliative care. J Pain Symptom Manag.

[7] Miyazaki K, Suzukamo Y, Shimozuma K, Nakayama T (2012). Verification of the psychometric properties of the Japanese version of the European Organization for Research and Treatment of Cancer quality of life questionnaire Core 15 palliative (EORTCQLQ-C15-PAL). Qual Life Res.

[8] Leppert W, Majkowicz M (2013). Validation of the polish version of the European Organization for Research and Treatment of Cancer quality of life questionnaire–Core 15–palliative care in patients with advanced cancer. Palliat Med.

[9] Nunes NAH (2014). The quality of life of Brazilian patients in palliative care: validation of the European Organization for Research and Treatment of Cancer quality of life questionnaire Core 15 PAL (EORTC QLQ-C15-PAL). Support Care Cancer.

[10] Alawneh A, Yasin H, Khirfan G, Qayas BA, Ammar K, Rimawi D, Klepstad P (2016). Psychometric properties of the Arabic version of EORTC QLQ-C15-PAL among cancer patients in Jordan. Support Care Cancer.

[11] Zhang L, Wang N, Zhang J, Liu J, Luo Z, Sun W, Woo SM, Chen C, Zhang K, Miller AR (2016). Cross-cultural verification of the EORTC QLQ-C15-PAL questionnaire in mainland China. Palliat Med.

[12] Ozcelik H, Guzel Y, Sonmez E, Aksoy F, Uslu R (2016). Reliability and validity of the Turkish version of the EORTC QLQ–C15–PAL for patients with advanced cancer. Palliat Support Care.

[13] Golčić M, Dobrila-Dintinjana R, Golčić G, Pavlović-Ružić I, Stevanović A, Gović-Golčić L (2018). Quality of life in a hospice: a validation of the Croatian version of the EORTC QLQ-C15-PAL. Am J Hosp Palliat Med.

[14] Drobnik J, Błaszczyk Feliks B, Nowak Piotr N, Beck Bogusław B, Susło R (2017). Comparison of the practical usefulness of the EORTC QLQ-C15 PAL and QLQ-C30 questionnaires on the quality of life of patients with pancreatic adenocarcinoma: estimation–preliminary study report. J Family Med Prim Care.

[15] Suárez-del-Real Y, Allende-Pérez S, Alférez-Mancera A, Rodríguez RB, Jiménez-Toxtle S, Mohar A, Oñate-Ocaña LF (2011). Validation of the Mexican–Spanish version of the EORTC QLQ-C15-PAL questionnaire for the evaluation of health-related quality of life in patients on palliative care. Psychooncology.

[16] Arraras JI, de la Vega FA, Asin G, Rico M, Zarandona U, Eito C, Cambra K, Barrondo M, Errasti M, Verdún J (2014). The EORTC QLQ-C15-PAL questionnaire: validation study for Spanish bone metastases patients. Qual Life Res.

[17] Irarrázaval ME, Rodríguez PF, Fasce G, Silva FW, Waintrub H, Torres C, Barriga C, Fritis M, Marín L (2013). Calidad de vida en cáncer de mama: validación del cuestionario BR23 en Chile. Rev Med Chil.

[18] Carcamo M, Campo V, Behrmann D, Celedón C, Alvear Á, Vásquez P, Araya C (2018). Cáncer de cabeza y cuello: validación de cuestionario QLQ-H&N35. Rev Med Chil.

[19] Urrutia MT, Concha X, Padilla O (2014). Calidad de vida en mujeres con cáncer cérvicouterino. Rev Chil Obstet Ginecol.

[20] Arancibia H, Carvajal C, Bustamante M, Justiniano JC, Talhouk O, Guler K, López I, Núñez J, Medina S (2009). Análisis de calidad de vida en pacientes gastrectomizados por cáncer gástrico. Rev Med Chil.

[21] Torres Ch P, Fasce G, Urrejola R, Pierotic M, León H, McConell Y, Urrejola L, Jiménez P, Yudin T, Carmona L (2010). Calidad de vida en pacientes con cáncer de cuello uterino: experiencia FALP. Rev Chil Obstet Ginecol.

[22] Irarrázaval ME, Kleinman P, Silva F, Fernández González L, Torres C, Fritis M, Barriga C, Waintrub H (2016). Calidad de vida en pacientes chilenas sobrevivientes de cáncer de mama. Rev Med Chil.

[23] Pastrana T, De Lima L, Wenk R, Eisenchlas J, Monti C, Rocafort J, Centeno C (2012). Atlas of palliative care in Latin America ALCP.

[24] Lynch T, Connor S, Clark D (2013). Mapping levels of palliative care development: a global update. J Pain Symptom Manag.

[25] Aaronson NK, Ahmedzai S, Bergman B, Bullinger M, Cull A, Duez NJ, Filiberti A, Flechtner H, Fleishman SB (1993). Haes JCJMd *et al*: the European Organization for Research and Treatment of Cancer QLQ-C30: a quality-of life instrument for use in international clinical trials in oncology. J Natl Cancer Inst.

[26] Fayers PM, Aaronson NK, Bjordal K, Groenvold M, Curran D, Bottomley A, On behalf of the EORTC Quality of Life Group (2001). the EORTC QLQ-C30 scoring manual.

[27] Johnson C, Aaronson N, Blazeby J, Bottomley A, Fayers P, Koller M, Kuliś D, Ramage J, Sprangers M, Velikova G (2011). EORTC quality of life group guidelines for developing questionnaire modules.

[28] Teunissen SC, Wesker W, Kruitwagen C, de Haes HC, Voest EE, de Graeff A (2007). Symptom prevalence in patients with incurable cancer: a systematic review. J Pain Symptom Manag.

[29] Moens K, Higginson IJ, Harding R, Brearley S, Caraceni A, Cohen J, Costantini M, Deliens L, Francke AL, Kaasa S (2014). Are there differences in the prevalence of palliative care-related problems in people living with advanced cancer and eight non-cancer conditions? A systematic review. J Pain Symptom Manag.

[30] Strömgren AS, Groenvold M, Pedersen L, Olsen AK, Sjogren P (2002). Symptomatology of cancer patients in palliative care: content validation of self-assessment questionnaires against medical records. Eur J Cancer.

[31] Arriaza P, Cancino G, Sanhueza O (2009). Pertenecer a algo mayor: experiencias de pacientes y cuidadores durante el cuidado paliativo en Chile. Cienc enferm.

[32] Ministerio de Salud, Gobierno de Chile (2011). Guía clínica AUGE “Alivio del Dolor por cáncer avanzado y Cuidados Paliativos”. Series Guías Clínicas MINSAL, 2011*.* Subsecretaría de Salud Pública, División de Prevención y Control de Enfermedades, Secretaría Técnica AUGE.

[33] Osman H, Shrestha S, Temin S, Ali ZV, Corvera RA, Ddungu HD, Lima LD, Estevez-Diz MDP, Ferris FD, Gafer N (2018). Palliative care in the global setting: ASCO resource-stratified practice guideline. J Glob Oncol.

