# Correction to: Content validation of the EORTC QLQ-C15-PAL with advanced cancer patients and health care professionals from palliative care services in Chile

**DOI:** 10.1186/s12904-020-00656-4

**Published:** 2020-10-12

**Authors:** Leslye Rojas-Concha, Maiken Bang Hansen, Morten Aagaard Petersen, Mogens Groenvold

**Affiliations:** 1 The Palliative Care Research Unit, Department of Geriatrics and Palliative Medicine GP, Bispebjerg and Frederiksberg Hospital, University of Copenhagen, Bispebjerg Bakke 23, DK-2400 Copenhagen, NV Denmark; 2 Department of Public Health, University of Copenhagen, Copenhagen, Denmark

**Correction to: BMC Palliat Care 19, 81 (2020)**

**https://doi.org/10.1186/s12904-020-00586-1**

Following publication of the original article [1], the authors identified an error in Table 3. The footnotes were missing. Provided below is the correct Table with its footer.

**Table 3:** Categorized reasons why some items were rated as little or not relevant by the participants

Scale/item	Item	Relevance^a^		Technical issues^b^		Inappropriate^c^		Relative^d^		Not important^e^		Difficult^f^		Total
		Pts.	HCPs	Pts.	HCPs	Pts.	HCPs	Pts.	HCPs	Pts.	HCPs	Pts.	HCPs	
Pain	9				1									1
	19		3		1		2	1						7
Physical functioning	1	5	5			3	3	1						17
	2	4	6	1	1	1		1		1	1			16
	3	4	6	1	2	1	1	1		1				17
	4	3				2	1	1					1	8
	5													–
Emotional functioning	21	1	5	1	2		1		1		1	1		13
	22	1	5	2	1			1		1	1			12
	23		1	4	5			1			1			12
	24		2		3			1						6
Fatigue	10		5		1		1	1		1	1			10
	12		3	2	3		1	2					1	12
	18	1	4	1			1	2			1			10
Nausea and vomiting	14	1	2		2									5
	15		2		1									3
Sleeping difficulties	11				1									1
Social functioning	26	1				1	1	1			1			5
	27		1		2	2		1			2			8
Dyspnea	8	3	5	14	13	1		1		1		1		39
Role functioning	6	2	1		3						1			7
	7	4	11		1	1	2	1						20
Constipation	16		1	10	7									18
Lack of appetite	13		1											1
Financial difficulties	28	1	2		1	2	1					1		8
Global health status/	29		2	1									1	4
Quality of life	30		1	2	3		1	1				1	1	10
Cognitive functioning	20	4	11		1		1				4			21
	25	1	8		1				1					11
Diarrhea	17		3			1			1					5
Total		36	96	39	56	15	17	18	3	5	14	4	4	307

^a^ Relevance: statements concerning the degree of relevance, i.e., replicating the quantitative data

^b^ Technical issues: statements that the question is not well formulated, reiterative or suggestions to combine items

^c^ Inappropriate: statements that the item was inappropriate

^d^ Relative: statements that the symptom or problem asked in the item depend of patient’s diagnosis

^e^ Not important: statements that the item was not important in palliative care

^f^ Difficult: the item was seen has difficult to understand

Patients: Pts., health care professionals: HCPs
